# MET Inhibitors in Small Cell Lung Cancer: From the Bench to the Bedside

**DOI:** 10.3390/cancers11101404

**Published:** 2019-09-20

**Authors:** Max Hardy-Werbin, Raúl del Rey-Vergara, Miguel Alejandro Galindo-Campos, Laura Moliner, Edurne Arriola

**Affiliations:** 1 Cancer Research Program, IMIM (Institut Hospital del Mar d’Investigacions Mèdiques), 08003 Barcelona, Spain; mhardy@imim.es (M.H.-W.); rdelrey@imim.es (R.d.R.-V.); mgalindo@imim.es (M.A.G.-C.); 2 Medical Oncology Department, Hospital del Mar-CIBERONC, 08003 Barcelona, Spain; lmoliner@psmar.cat

**Keywords:** small cell lung cancer, MET, HGF, immunotherapy

## Abstract

Small cell lung cancer (SCLC) is the most aggressive type of lung cancer. The different systemic treatment approaches attempted in the last 35 years have not improved overall survival in the advanced stage. Targeted therapies assessed in clinical trials have failed to show efficacy against SCLC. Within the potentially interesting targets, the hepatocyte growth factor (HGF)/mesenchymal-epithelial transition (MET) pathway activation is associated with worse survival and chemoresistance in SCLC. Preclinical data suggest that the inhibition of the MET pathway can revert chemoresistance and prevent tumor growth. Recently, immunotherapy has shown modest but relevant activity in SCLC. Interestingly, MET modulation seems to be involved in increasing the efficacy of standard checkpoint inhibitors. Here, we review the preclinical and clinical data of MET inhibition in SCLC, and the role of this pathway in the immune response.

## 1. Introduction

Lung cancer is the most common cancer worldwide [1]. Its incidence has increased over the years [2], and it remains the leading cause of cancer-related deaths [3]. Out of all lung cancer patients, approximately 13% are diagnosed with small cell lung cancer (SCLC) [4,5].

SCLC is the most aggressive type of lung cancer and it is highly linked to tobacco smoking [6,7]. Combination treatment with chemotherapy and radiotherapy in a limited stage has increased survival rates [8]. However, more than half of SCLC patients are diagnosed at an extensive stage, where treatments are scarce, leaving SCLC as an orphan disease with a dismal prognosis [9]. In fact, platinum-based chemotherapy has been the systemic standard treatment in an extensive disease since the mid-1980s [10,11]. Although substantial clinical responses are achieved after first-line treatment, disease progression takes place quickly and it is usually resistant to available treatments. In this scenario, SCLC presents with a 5-year overall survival (OS) of around 6% [12], dropping to 3% [4] if only the extensive disease population is considered. Thus, there is an urgent need for the development of effective therapies in SCLC. 

The emergence of immunotherapy has represented the first shift in decades in the treatment of advanced SCLC, and checkpoint inhibitors have been recently approved by the Food and Drug Administration (FDA) for the treatment of this disease. Based on the results of the phase I/II trial CheckMate 032, the combination of nivolumab and ipilimumab was added as a recommendation in the National Comprehensive Cancer Network (NCCN) guidelines, and afterwards, nivolumab, as single-agent, was approved by the FDA in the third-line setting [13]. Moreover, in a recently published clinical trial (IMpower 133), the addition of atezolizumab to standard chemotherapy showed benefit in terms of overall survival in chemo-naive SCLC patients [14]. Nevertheless, only a small subset of unselected patients seems to benefit from immunotherapy. Despite several biomarkers that have been evaluated as predictive markers showing benefit from immunotherapy in SCLC [15,16], these have not yet been validated.

SCLC is characterized as having one of the highest rates of mutational tumor burden [17]. However, despite the multiple genomic aberrations found in SCLC [18], these are rarely druggable. Although many attempts have been made, targeted therapies have not shown benefits regarding SCLC treatment [19,20]. One of the potentially interesting targets, linked to mechanisms of chemoresistance in SCLC, is the activation of the hepatocyte growth factor (HGF)/mesenchymal-epithelial transition (MET) receptor tyrosine kinase (RTK) signaling pathway via the induction of the epithelial to mesenchymal transition (EMT). In preclinical models it has been shown that this pathway plays a key role in the aggressiveness of SCLC [21] and that its inhibition leads to the reversion of chemoresistance and tumor response [22]. This review discusses the role of the HGF/MET pathway in SCLC and the potential therapeutic value of MET inhibition in this deadly disease.

## 2. Description of the MET Molecule and the Downstream Pathway

The human proto-oncogene *MET* was originally described in 1984 [23]. After treating a human osteosarcoma cell line with a chemical carcinogen (N-methyl-N0-nitro-N-nitrosoguanidine), a fusion protein with the translocated promoter region (TPR) and the MET domain was obtained. The TPR region of the protein induced a constitutive dimerization, which led to an activation of the MET kinase domain. *MET* is located in chromosome 7q21-q31 [24], and codifies to a high-affinity transmembrane RTK [25]. The MET receptor is a disulphide linked glycoprotein heterodimeric RTK. The structure of the molecule consists of an extracellular α-chain linked to transmembrane-spanning β-chain. The α-chain and the first fragments of the β-chain are responsible for the binding to its ligand, the HGF, while the intracellular region of the β-chain is responsible of the downstream signaling [26]. 

By the time the MET receptor was characterized, its ligand had not yet been identified [27]. Later, HGF was described by two independent groups as a motility and a mitogenic factor [28,29], and then defined as the MET ligand [30]; nowadays, it remains as the sole identified ligand of the MET receptor.

After binding to HGF, the MET receptor suffers a dimerization [31], which causes a phosphorylation in the tyrosine residues of the cytoplasmic domain, mainly at phospho-epitopes pY1230, pY1234, and pY1235 [32,33]. Afterwards, phosphorylation in pY1349 promotes the recruitment of several substrates, such as growth factor receptor-bound protein 2 (Grb2), Gab1 (Grb2-associated-binding protein 1), Src and phospholipase C [34], that are responsible for the following downstream signaling, essentially through the rat sarcoma (RAS)-mitogen-activated protein kinase (MAPK) and phosphoinositide 3-kinase (PI3K)-protein kinase B (AKT) pathways [26,32,35,36]. 

Under physiological conditions, the HGF/MET pathway mediates several cellular responses, such as cell migration [36] and tissue regeneration [37,38]. In this context, MET activation is basically controlled in two different ways: 1) internalization by endocytosis into multivesicular bodies, followed by ubiquitination and degradation [39,40]; and 2) fading of signaling due to the action of specific phosphatases [41]. 

**Figure 1:**
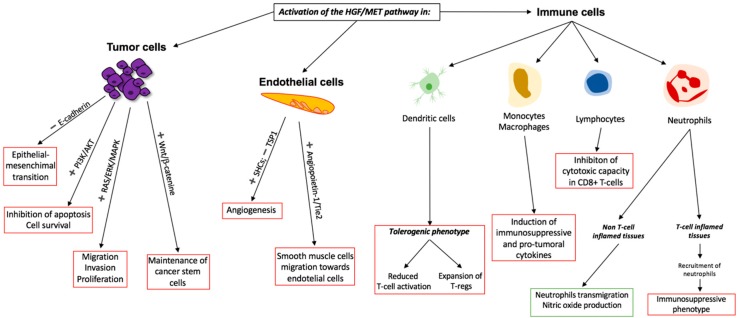
Role of the activation of the HGF/MET axis in tumor cells, endothelial cells, and immune cells. Red boxes define pro-tumorogenic functions. Green boxes define anti-tumorogenic functions. PI3K, phosphoinositide 3-kinase; ERK, extracellular signal-regulated kinase; MAPK, mitogen-activated protein kinase; SHCs—SRC homology 2 domain-containing proteins; TSP1—thrombospondin 1; Tie2—angiopoietin receptor 2.

However, aberrant activation of the MET pathway leads to increased cell motility and cell cycle progression and many other protumorigenic signaling (Figure 1), and has been proven to be involved in many types of cancer, as discussed below.

## 3. Role of the HGF/MET Axis in Cancer

During the last three decades, the dysregulation of the HGF/MET pathway has been identified as a common characteristic in different types of human cancer. In the early 1990s, Rong et al. proved the tumorigenic feature of the oncogene *MET* and the metastatic potential of the overexpression of HGF in murine and human cancer cell lines [42,43]. This fact was confirmed by a loss of aggressiveness when the HGF/MET axis was downregulated in human tumors [44]. Later on, many studies showed that HGF and MET were often expressed in a large variety of cancers [45,46,47,48,49,50]. 

The MET downstream signaling can be aberrantly enhanced by gene mutation or amplification, by protein overexpression, or in a ligand-dependent fashion. In 1997, Schmidt et al. [51] reported, for the first time, activating mutations of MET in renal cancer. Further studies found other MET mutations in many cancer types, including SCLC [52,53], supporting the implication of this receptor in human cancer [54,55,56,57,58]. Some of these mutations were later identified as non-pathogenic, thus more research is required in this field [21].

Among the acquired functions due to the aberrant activation of MET, the epithelial-mesenchymal transition (EMT) is of pronounced significance. EMT is a phenomenon whereby cancer cells suffer a loss of epithelial features and acquire a mesenchymal phenotype, conferring the capability to go through the boundaries of the tumor and metastasize [59]. The driver characteristic of this process is the suppression of E-cadherin, which is mainly induced by the expression of the transcriptional factor Snail. It has been reported that HGF is an effective inductor of EMT [60,61]. Our group has previously demonstrated that activation of the HGF/MET pathway induced an EMT phenotype in a SCLC preclinical model [21]. Moreover, MET inhibition was able to revert the mesenchymal features on these cells.

The HGF/MET axis also plays a crucial role in resistance to targeted drugs. It has been show that HGF can induce tyrosine-kinase inhibitors resistance in lung adenocarcinoma harboring epidermal growth factor receptor (EGFR) mutations, presumably through cross-talk activation of the PI3K/AKT pathway [62,63].

In addition to its role in tumor cells, MET activation can also be observed in endothelial cells, promoting angiogenesis [64,65], thus contributing to tumor progression. Through the regulation of diverse intermediates, such as SHCs (SRC homology 2 domain-containing proteins) and TSP1 (thrombospondin), MET favors the induction of endothelial cell growth [66] (Figure 1). 

Activation of the HGF/MET axis has not only been shown to be a marker of aggressiveness in preclinical research. Several groups reported a decreased survival rate in cancer patients with an aberrant tumor expression of either MET or its ligand HGF, including SCLC patients [67,68,69,70].

## 4. MET in SCLC

### 4.1. Emergence of MET in the SCLC Biomarker Spectrum

The first study to show the expression of MET in SCLC was conducted by Rygaard et al. in 1993 [71]. They examined a panel of 25 SCLC cell lines and xenografts, demonstrating the presence of MET mRNA transcripts and protein expression, but failing to show that these same tumor cells co-expressed its ligand, HGF. 

The role of the MET/HGF axis in SCLC was later elucidated via stimulation of SCLC cell lines with HGF. Maulik et al. found an increased tyrosine phosphorylation of MET, and an increase in cell motility (observed by the formation of filopodia) in response to HGF [72]. They also showed that the heat shock protein 90 (Hsp90) inhibitor geldanamycin, a known inhibitor of the HGF/MET pathway [73], increased apoptosis, and decreased motility and MET expression in SCLC cell lines. Further research supported this concept by showing that the C-terminus of the Hsp70-interacting protein (CHIP), which also targets Hsp90, is essential for MET regulation by blocking cell survival pathways in SCLC cell-lines [74]. Similar effects were seen when inhibiting PI3K, a downstream factor of MET, in MET-dependent SCLC cell lines [75]. 

### 4.2. Small MET Inhibitor Molecules

In light of the relevance of the HGF/MET axis activation in SCLC, several novel MET inhibitors were tested. SU11274 is a small molecule inhibitor of MET and it has been proved to be 50 times more selective for MET relatively to other kinases [76]. SU11274 selectively inhibited MET tyrosine kinase activity in a cell line transformed by the oncogenic TPR-MET protein (a feature also present in SCLC), inducing cell cycle arrest and apoptosis [77]. This inhibition was specifically replicated in non-small-cell lung cancer NSCLC and SCLC cell lines and supported by obtaining the same results after the inhibition of MET via knock-down with a small interference RNA (siRNA) [33,78]. 

MET targeting drugs were later tested in vivo. PHA665752 is a specific, small-molecule MET ATP-competitive inhibitor that also synergizes with the downstream specific mTOR inhibitor rapamycin [79]. SCLC xenograft mouse models treated with PHA665752 showed high rates of tumor response [80]. Of note, these mice were injected with the drug intratumorally. Further in vitro assays with SCLC cell lines were able to support the concept regarding how PHA665752 counteracts the stimulation of HGF in *MET* mutant SCLC [22].

### 4.3. Combination of MET Inhibitors with Chemotherapy

Once specific MET inhibitors were shown to be active in SCLC, further new strategies were evaluated. Prof. Salgia’s group [81] aimed to look for combination strategies with chemotherapeutic agents and MET inhibitors. They found that extensive SCLC patients overexpressed nuclear topoisomerase-1, and that this fact was correlated with the increased expression of MET. Thus, they tested the MET inhibitor SU11274 in vitro in combination with 7-Ethyl-10-hydroxy-camptothecin (SN-38), the active metabolite of irinotecan [82], a topoisomerase inhibitor used in refractory disease in SCLC [83]. Both compounds combined synergized together, achieving a greater decrease on cell viability in the SCLC cell line H69 than when used alone. 

Shortly after, a multikinase inhibitor was tested by our group [21] in combination with the topoisomerase-2 inhibitor etoposide. PF-2341066 (crizotinib), a small molecule inhibitor, showed activity against the anaplastic lymphoma RTK (ALK) and MET [84]. In order to establish the role of the MET/HGF axis activation in chemoresistance, we were able to resensitize chemoresistant SCLC cell lines, both in vitro and in vivo after treatment with PF-2341066. Moreover, a significant decrease in tumor growth was observed on H69M (mesenchymal cells derived from H69 after HGF exposure) xenografted mice after treatment with PF-2341066 combined with etoposide [21]. These findings were subsequently validated by different groups. Ozasa et al. [85] reported that treating SCLC chemoresistant cell lines with the MET inhibitor SU11274 combined with irinotecan reverted the resistance to cytotoxic treatment. In a preclinical scenario from an orthotopic SCLC model with extensive disease, Sakamoto et al. showed that treatment with the Met inhibitor PHA665752 inhibited motility and the invasion of SCLC cells harboring high HGF expression [86]. In this case, combination with chemotherapy was not assessed. In a more recent work, Taniguchi et al. [87] also showed similar results, both in vitro and in vivo, with E7050 (golvatinib), a dual kinase inhibitor of MET and vascular endothelial growth factor receptor-2 (VEGFR-2), which is currently under clinical development [88]. Growth of SCLC cell lines expressing HGF and phosphorylated-MET (confirmed using Western blot and Enzyme-linked immunosorbent assay) was constrained after in vitro treatment with golvatinib. Moreover, this MET inhibitor was able to avoid systemic SCLC metastases in vivo. In this study, the proof of concept was supported by obtaining similar results with a knockdown of HGF and MET. Of note, the same results were observed using crizotinib. Combinations with chemotherapeutic agents was not evaluated. These data suggest a role for MET inhibition in SCLC (Table 1).

### 4.4. Clinical Trials

In spite of all the preclinical research of the efficacy of anti-MET therapy in SCLC developed in the last two decades, few clinical trials have been conducted, of which none delivered positive results. 

Tivantinib (ARQ-197) is a small-molecule inhibitor of MET [89]. However, its main mechanism of action may involve a tubulin depolymerization [90]. It has been tested in a phase I clinical trial [91], in combination with topotecan, in patients with advanced solid tumors, including three patients with SCLC. An expansion cohort consisting of advanced stage SCLC was planned, but development was halted due to poor tolerability of the combination. Moreover, no responses were seen. During the course of this trial, a concomitant phase II clinical trial with tivantinib as a maintenance treatment in advanced stage SCLC was initiated, but suffered an early termination due to the preliminary safety results [92].

Efficacy of rilotumumab (anti-HGF) or ganitumab (anti-insulin-like growth factor 1 receptor) in combination with platinum-etoposide was assessed in first-line treatment in advanced stage SCLC. A phase Ib/II clinical trial [93] failed to show improved outcomes with the addition of an anti-HGF monoclonal antibody to chemotherapy. This was a two-part study: the first part was a dose escalation phase for both inhibitors; and the second part was a randomized placebo-controlled phase using the doses identified in the first part where patients were assigned to receive rilotumumab, ganitumab, or placebo, plus chemotherapy. The overall survival in the anti-HGF group was higher than the placebo group (12.2 vs 10.8 months), although this difference was not statistically significant. Objective response rate (ORR) was 59% in the placebo group and 68% in the rilotumumab group.

Finally, a phase II clinical trial [94] evaluating the efficacy of amuvatinib, a multi-targeted tyrosine kinase inhibitor, in combination with platinum-etoposide in relapsed SCLC patients was conducted. Although the study was designed mainly because of the activity of the inhibitor in c-KIT mutant forms, it also has activity against MET. However, the trial failed to meet its primary objective, which discouraged further development of the drug in this scenario.

### 4.5. Prevalence of MET Alterations in SCLC

The data available to this day regarding the importance of aberrant activation of the HGF/MET axis in SCLC supports the evaluation of the inhibition of this pathway as a therapeutic strategy. Initially, Ma et al. reported a prevalence of 12.5% in a cohort of 32 SCLC tumor samples [52]: only one mutation (E168D) was shown to elicit a gain of functions. Later on, Pfeifer et al. did an integrative genome analyses in 99 SCLC samples and did not find any MET pathogenic mutation [95]. More recently, two studies reported a MET mutation prevalence of 6.5% and 4.4% in 46 and 113 SCLC tumor specimens, respectively [96,97]. Globally, the prevalence of MET mutations in SCLC varies among the different studies, and most of the mutations were found to be non-pathogenic. Other mechanisms, such as increased HGF secretion, might identify a subset of patients eligible for effective MET inhibition [21]. Interestingly, a recent publication highlights the relevance of selective recognition and imaging of two-chain active HGF using a macrocyclic peptide that might serve as a biomarker for MET-activated tumors targetable with MET inhibitors [98]. The complexity of the MET receptor ligand’s dependent and independent activation will require comprehensive biomarker studies when testing MET inhibitors in this disease.

## 5. Rationale for MET Inhibition in Combination with Immunotherapy

There is increasing evidence of the role of the HGF/MET signaling in immune responses, and its potential effects in the immune compartment of the tumor microenvironment (Figure 1). 

As mentioned before, MET is highly expressed in many cancer types, acting as an epitope. As such, it can be recognized by cytotoxic CD8+ T cells, eliciting an activation toward tumor cells expressing MET [99]. 

On the other hand, MET is not only expressed by cancer cells, but also in stromal and immune cells [100,101]. It plays a crucial role involving dendritic cells (DCs). MET activation through HGF stimulation in DCs may lead to a tolerogenic phenotype with the consequent proliferation of regulatory CD4+ T cells and a decrease of cytotoxic CD8+ T cells, resulting in immunosuppression [102]. In the T cell compartment, it has been recently reported that a fraction of CD8+ T cells express MET, and that upon stimulation with HGF, their cytotoxic capacity is restrained [103].

MET is also expressed in neutrophils. Finisguerra et al. [104] showed that MET deletion in neutrophils in a murine model enhances tumor growth and metastasis. This suggests that MET constitutes a relevant requirement for neutrophils chemoattraction and cytotoxicity in order to trigger an anti-tumor local immune response, mainly through an HGF/MET-dependent cytotoxic nitric oxide release. These findings might be relevant when treating SCLC with MET inhibitors regardless of MET status, in which the neutrophil mediated cytotoxicity may play a significant role.

Even though these findings call for caution, the scenario changes when immunotherapy (anti-programmed death(PD)-1 agents) is added into the treatment regime. In a tumoral context, interleukin (IL)-17 from γδ T cells induce neutrophils’ ability to suppress cytotoxic CD8+ T cells [105]. Glodde et al. [106] showed in immune-competent mice treated with anti-PD-1, that neutrophils recruited to the tumor acquired an immunosupressive phenotype upon exposure to interferon (IFN)γ, which was mainly released by activated T cells in the tumor microenvironment. The addition of a MET inhibitor to immunotherapy blocked the reactive migration of neutrophils from the bone marrow to the tumor, achieving better responses to anti-PD-1 agents. These results were replicated in mice with tumors harboring MET aberrant signaling, as well as in mice with wild-type MET, reinforcing the potential efficacy of a combination strategy of immunotherapy and anti-MET inhibitors, regardless of the tumor MET status.

There is recent evidence suggesting that patients with MET exon 14 mutant lung cancer respond worse to immunotherapy [107,108], further supporting exploring the combination. However, preclinical and clinical data evaluating the combination of immunotherapy and MET inhibitors in SCLC are still lacking.

## 6. Conclusions

SCLC is a highly lethal malignancy with a dismal prognosis and little improvements have been achieved to date. In the last few years, the advent of immunotherapy has allowed us to witness the emergence of a small percentage of patients presenting a longer survival than expected. However, targeted therapies failed to show any progress and none of them are available for the treatment of patients with SCLC.

There are currently around 120 clinical trials in active recruitment worldwide involving targeted therapies and immunotherapy in SCLC, including those basket trials for solid tumors, allowing for the inclusion of SCLC patients. Of those, none of them includes drugs targeting MET to our knowledge. 

In the past, only two trials attempted to block MET with a selective target SCLC population, and both were negative. Diverse explanations can be hypothesized for this failure. First, the anti-MET agents used had scarce preclinical data on SCLC. Rilotumumab (AMG-102) is a monoclonal antibody that prevents the HGF binding to the MET receptor; to the best of our knowledge there are no reports on preclinical data of rilotumumab in SCLC. Furthermore, given the consecutive negative results in several clinical trials and its increased toxicity, its mechanism of action was further studied. Greenall et al. [109] reported that rilotumumab does not prevent HGF from directly binding to HGF, which maybe explains the negative trial results. Tivantinib (ARQ-197), a selective non-ATP-competitive inhibitor of MET, also failed to demonstrate efficacy in SCLC, and it showed intolerable toxicity when combined with chemotherapy. Preclinical data were lacking evaluating activity of this molecule in MET-dependent SCLC models [110]. Furthermore, it has been reported that its efficacy is independent of MET inhibition due to its primary mechanism of action via tubulin depolymerization [90], which highlights the relevance of thorough preclinical and mechanistic studies before initiating a clinical trial. Notably, MET status in patients recruited in these clinical trials had not been assessed, leading one to suspect that the population was not properly selected and therefore may mask the benefit for MET driven tumors.

Immunotherapy has shifted how cancer patients are treated nowadays, and SCLC patients are no exception. A combination of immunotherapy and MET-inhibitors seems a potentially promising strategy to be assessed regarding SCLC, irrespective of patients harboring MET overexpression/mutation. Adequate selection of patients for clinical trial success will require biomarker studies including MET activation, HGF expression, immune infiltrate evaluation, and others.

In conclusion, we believe there is rationale for the evaluation of MET inhibitors with immunotherapy in SCLC. It is now time to design proper clinical trials and take this knowledge from the bench to the bedside. 

## Figures and Tables

**Table 1:** Preclinical testing of anti-MET targeted therapy in SCLC.

Anti-MET Drug	Regimen	Reference	Test Setting
NSC122750 (geldanamycin)	Monotherapy	Maulik et al. (2002) [64]	In vitro
SU11274	Monotherapy	Sattler et al. (2003) [69] Ma et al. (2007) [31] Ma et al. (2005) [70]	In vitro In vitro In vitro
SU11274	Combination with irinotecan	Rolle et al. (2013) [74] Osaza et al. (2014) [78]	In vitro In vitro
PHA665752	Monotherapy	Puri et al. (2007) [72] Arriola et al. (2011) [20] Sakamoto et al. (2015) [79]	In vivo In vitro In vitro
PF2341066 (criozotinib)	Combination with etoposide	Cañadas et al. (2014) [19] Taniguchi et al. (2017) [80]	In vitro/in vivo In vitro/in vivo
E7050 (golvatinib)	Monotherapy	Taniguchi et al. (2017) [80]	In vitro/in vivo
